# Bovine Mastitis: Frontiers in Immunogenetics

**DOI:** 10.3389/fimmu.2014.00493

**Published:** 2014-10-07

**Authors:** Kathleen Thompson-Crispi, Heba Atalla, Filippo Miglior, Bonnie A. Mallard

**Affiliations:** ^1^Department of Pathobiology, University of Guelph, Guelph, ON, Canada; ^2^Center for Genetic Improvement of Livestock, University of Guelph, Guelph, ON, Canada; ^3^Department of Biomedical Science, University of Guelph, Guelph, ON, Canada; ^4^Department of Animal and Poultry Science, University of Guelph, Guelph, ON, Canada; ^5^Canadian Dairy Network, Guelph, ON, Canada

**Keywords:** disease resistance, genetic selection, genomics, immune response, mastitis

## Abstract

Mastitis is one of the most prevalent and costly diseases in the dairy industry with losses attributable to reduced milk production, discarded milk, early culling, veterinary services, and labor costs. Typically, mastitis is an inflammation of the mammary gland most often, but not limited to, bacterial infection, and is characterized by the movement of leukocytes and serum proteins from the blood to the site of infection. It contributes to compromised milk quality and the potential spread of antimicrobial resistance if antibiotic treatment is not astutely applied. Despite the implementation of management practises and genetic selection approaches, bovine mastitis control continues to be inadequate. However, some novel genetic strategies have recently been demonstrated to reduce mastitis incidence by taking advantage of a cow’s natural ability to make appropriate immune responses against invading pathogens. Specifically, dairy cattle with enhanced and balanced immune responses have a lower occurrence of disease, including mastitis, and they can be identified and selected for using the high immune response (HIR) technology. Enhanced immune responsiveness is also associated with improved response to vaccination, increased milk, and colostrum quality. Since immunity is an important fitness trait, beneficial associations with longevity and reproduction are also often noted. This review highlights the genetic regulation of the bovine immune system and its vital contributions to disease resistance. Genetic selection approaches currently used in the dairy industry to reduce the incidence of disease are reviewed, including the HIR technology, genomics to improve disease resistance or immune response, as well as the Immunity^+^™ sire line. Improving the overall immune responsiveness of cattle is expected to provide superior disease resistance, increasing animal welfare and food quality while maintaining favorable production levels to feed a growing population.

## Introduction

Mastitis, generally defined as the inflammation of the mammary gland, is a costly and complex disease associated with variable origin, severity, and outcome depending on the environment, pathogen, and host (1, 2). Mastitis is caused when pathogenic bacteria enter the sterile environment of the mammary gland, often as a result of disruption of physical barriers such as the teat, requiring prompt and appropriate host defenses to prevent colonization and subsequent disease pathology (3, 4). Mastitis-causing pathogens are commonly categorized as environmental or contagious, although this distinction has recently been disputed (5). Nonetheless, in general environmental pathogens have been grouped to include coliforms like *Klebsiella* or *Escherichia coli* (*E*. *coli*) and streptococci and are a major cause of clinical mastitis. On the other hand, those categorized as contagious pathogens can readily be spread from the infected quarters to other quarters of the same cow, or other cows and include *Staphylococcus aureus* (*S*. *aureus*) and *Streptococcus agalactiae* (6–8). Cow factors including age, stage of lactation, and somatic cell score (SCC) history are known to influence the occurrence of mastitis infection (9, 10). The diverse pathogens that can cause mastitis induce different immune responses in the mammary gland, and therefore, the host requires highly specific pathogen-dependent responses for protection (11, 12).

Mastitis infections are described as either subclinical or clinical. Subclinical mastitis is the presence of infection without local inflammation resulting in an absence of visual signs (1). It may involve transient cases of inflammation and abnormal milk, and if this persists for longer than 2 months is termed chronic. Clinical mastitis, on the other hand, is an inflammatory response causing visibly abnormal milk. In the case of mild or moderate clinical mastitis, changes in the udder may include swelling, heat, pain, and redness. It is termed severe if the response includes systemic involvement such as fever, anorexia, and shock (13, 14). The diversity as well as the variation in prevalence and abundance of mastitis-causing organisms as well as the variation in host responses make mastitis a complex disease that continues to be a burden for the dairy industry.

The bovine mammary gland is equipped with a non-immune anatomical barrier, and a plethora of immune-mediated defense mechanisms that include innate and adaptive immune responses. Innate immunity is relatively non-specific with rapid kinetics while the adaptive immunity offers a highly specific response with relatively delayed kinetics (15). Innate host-defenses depend on germline-encoded receptors that recognize conserved structures expressed by a wide range of microbes, and early induced cellular and soluble defenses. These natural defenses respond quickly to microbes during early stages of infection and are tightly integrated with the adaptive immune system. The innate host defenses of the mammary gland have been reviewed extensively elsewhere (16–18). The adaptive immune system uses a diverse repertoire of antigen specific receptors expressed by clonally expanded B and T-lymphocytes to regulate or eliminate the signal elicited by recognition events. Additionally, the induced adaptive immune response has the capacity to establish antigen specific memory for a rapid and augmented response upon subsequent exposure to the same antigen (19). For example, these various components of the immune system work in collaboration both locally and systemically in an attempt to control specific mastitis pathogens invading the mammary gland, but the details of the response is contingent upon the stage of infection and nature of the pathogen, as well as its interaction with the genetics of the host.

The interaction between mastitis pathogens and the host immune system is intricate, since both have the ability to co-evolve to recognize, respond, and adapt to the other. As such, microbial pathogens have developed various strategies to alter and evade host defenses in order to survive. Importantly, the host immune system is also adaptive and has a large arsenal to control or eliminate microbial threat. Even so, it is widely accepted that susceptibility of individuals within a given species differs to the same microbial pathogen. This variability in host–pathogen interaction is controlled by the inherent genetic make-up of the host, including innate and adaptive immune responses, particularly the acquired immunological memory, as well as the nature of the microbial pathogen (20).

Mastitis causing-bacterial pathogens are often well adapted to the bovine host resulting in clinical signs and, occasionally, subclinical infection before they lead to chronicity and persistence in the mammary gland. Persistent intramammary infections are frequently associated with recurrent clinical episodes and long-term increases in milk somatic cells counts. Persistent strains often express sets of genes that relate to their adaptation to the intramammary milieu and allow for intracellular survival and subsequent modulation of host-defense mechanisms (6, 21). *S. aureus* and *E*. *coli* are well-studied mastitis pathogens in the context of host–pathogen interaction and the elucidation of their genes, along with host immune response genes, is launching new studies in functional genomics (20). Understanding sequence data and locating functional SNPs in both the host and pathogen is expected to reveal relationships between immune function and the relevant genes that have the potential to advance resistance to specific pathogens.

Treatment of mastitis is given on the premise that treatment costs will be outweighed by production gains resulting from elimination of infection. Most farms have established mastitis management programs and include strategies such as routine whole herd antibiotic therapy, culling of chronically affected cows, post milking teat disinfection, as well as ensuring routine maintenance of milking machines (7, 14). Due to high treatment costs, lost income due to discarded milk, public health, and animal welfare concerns, it would be advantageous for dairy cattle to resist or mount effective immune responses to clear the wide variety of mastitis-causing pathogens. In the case of mastitis, the ability to control or tolerate the infection without actually clearing the pathogen, a phenomena known as resilience or tolerance (22), is not sufficient given that dairy products are consumed by human beings and are expected to be free of all potentially harmful pathogens. Antimicrobial treatment has the potential to increase the risk of antibiotic resistant strains of bacteria emerging in the environment (23), although it has been suggested that scientific evidence does not support emerging resistance in pathogens isolated from dairy cows (24). Nonetheless, other non-antibiotic treatment strategies are clearly warranted. Additionally, decreasing the incidence of mastitis would contribute to increased animal welfare as severe signs are associated with pain and discomfort for the cow (25).

Mastitis is a problem that plagues dairy cattle worldwide; however, this review will focus on the mastitis situation in the most economically developed countries. We highlight the genetic regulation of the bovine immune system and its vital contributions to disease resistance, in particular mastitis. Current genetic selection approaches used in the dairy industry to reduce the incidence of disease are reviewed, including the HIR technology; the Immunity^+^™ sire line, as well as genomics to improve disease resistance or immune response. While the complex interactions of the host and pathogen are fully acknowledged, they are only briefly discussed here.

## Genetic Regulation of the Immune System

Robust, appropriate and timely host defense mechanisms are critical for prompt bacterial clearance and prevention of mastitis and mammary epithelial damage (14). Bacteria have a large repertoire of virulence factors that are produced at varying concentrations depending on the stage of infection (26), and these virulence factors in part determine differences in the magnitude and duration of host immune responses. Further, given the diversity of mastitis-causing pathogens, it is essential for the host to have a broad range of host-defense mechanisms as part of its immunological arsenal. Both innate and adaptive host defenses are required to protect the host from infection. Innate defenses against mastitis pathogens are rapid and include neutrophil recruitment to the mammary gland to facilitate bacterial clearance through phagocytosis, production of reactive oxygen species, antibacterial peptides, such as lactoferrin and β-lactoglobulin, and defensins, resulting in increases in the somatic cell count (18, 27). Mammary epithelial cells are known to play a role in early responses through the production of cytokines like IL-8 and other factors with antimicrobial activities (28, 29). If the bacteria survive these innate host defenses, adaptive immune responses mediated by T and B cells are required to clear the infection (30). The ideal immune response being one that appropriately recognizes epitopes on the invading pathogen to initiate swift and accurate clearance mechanisms while maintaining minimal pathological consequences. In some situations, such as experiments using *in vitro* or *in vivo* lipopolysaccaride challenge to measure bovine inflammatory responses, particularly IL-8, have noted that cows with lower IL-8 responses had quicker recovery in terms of somatic cell counts and milk production than those with high IL-8 production (31). This may relate to a more moderate inflammatory response generated in these low IL-8 responders. However, it is important to note that this does not mean that cows classified as low responders for other immune response mechanisms, particularly adaptive immune responses are advantageous. In fact, dairy cows classified as high responders (robust and balanced responses) for adaptive immune response traits have been demonstrated to have reduced disease incidence (32). The other thing worth noting in these experiments was the observation that the differences between high and low IL-8 responses seemed to be controlled by epigenetic effects (33). Epigenetic influences on bovine type 1 (Interferon-γ) and type 2 (IL-4) cytokine production have also been reported in cows classified as high or low responders based on their antibody and cell-mediated immune responses (34). Researchers are only beginning to dissect both the genetic and epigenetic mechanisms that control immunity.

Initiation and regulation of adaptive immune responses are critical to the resolution of infection. Cells of the innate immune system recognize conserved pathogen associated molecular patterns from the bacteria by binding pattern recognition receptors on antigen-presenting cells (APC) such as macrophages and dendritic cells (35). Such pattern recognition receptors include toll-like receptors (TLR) that are located on cell and endosomal membranes (27, 36). The association of a TLR with a pathogen associated molecular pattern initiates a downstream signaling cascade leading to the activation of transcription factors, such as NF-κβ, which enter the nucleus, bind target promoters, and may induce the production of cytokines and other endogenous mediators. The 10 mammalian TLRs are known to elicit unique responses through intracellular signaling pathways, which initiate inflammatory and antimicrobial processes to eliminate the pathogen (36, 37). For example, the recognition of lipopolysaccharide (LPS) from *E. coli* by TLR4, facilitated by additional proteins including CD14, LPS binding protein, and myeloid differentiation protein, is associated with production of TNF-α, IL-1β, IL-6, and IL-8. The lipoteichoic acid of Gram positive bacteria like *S. aureus* recognized by TLR2 is associated with only transient increases in TNF-α and IL-1β as well as IgG2 (27). It is well recognized that *E. coli* induces a stronger increase in the pro-inflammatory cytokines TNF-α and IL-1β compared to *S. aureus* (12, 27, 38), contributing to the severe clinical signs typically associated with *E. coli* mastitis as compared to *S. aureus* where the majority of cases go unnoticed. This draws attention to the fact that although the innate immune responses provide a first line of defense against invading microbial pathogens, including those that cause mastitis, and contours ensuing adaptive immune responses; innate responses have the potential to generate harmful pathology by driving inappropriate or soaring inflammatory cascades (31). These need to be carefully considered and closely monitored when considering immunological interventions.

The major histocompatability complex (MHC) plays an essential role in the induction and regulation of immune responses (39). The bovine MHC, bovine lymphocyte antigen (BoLA), has been associated with resistance or susceptibility to mastitis (40–43), somatic cell count (42, 44, 45), and immune response (40, 41, 46). Genetic variation, such as single nucleotide polymorphisms (SNP) in other candidate genes associated with resistance or susceptibility to mastitis have been identified, including TLR4 (47, 48), TLR2, and caspase-recruitment domain 15 (49); IL-10 (50), osteopontin (51), IL-8 and its receptor CXCR1 (52–54), CCL2 and its receptor (55), as well as a variety of other genes (56). Other molecules important in host defense against mastitis-causing pathogens such as β-defensins have been identified and their complex genetic regulation is beginning to be understood (57). The feasibility of breeding for resistance based on one SNP or a combination of SNP depends on the degree of variation each SNP explains in resistance to mastitis. Since mastitis is a complex genetic trait a combination of many genes will ultimately be responsible for resistance to mastitis; however, certain major genes may contribute more benefit than others and it is important that these genes be elucidated.

Recent studies are beginning to uncover information about the epigenetic influences on bovine immune response genes (58). Some studies are now indicating that epigenetic changes are involved in the regulation of type I and II immune responses of mammals (59, 60), including cytokine profiles of dairy cows during the peripartum period when the risk of mastitis is the greatest (34). Epigenetic modifications have also been demonstrated to play a role in bovine innate immune responses to LPS stimulation (33, 61). Further, microRNA have been found to be differentially expressed upon challenge with mastitis-causing pathogens, suggesting a role for microRNA in regulating host responses to mastitis (62, 63). Indeed, many studies have demonstrated the bovine immune response to be under genetic and epigenetic control, and making use of this information in breeding strategies is anticipated to help improve udder health.

The important question is how to use this information regarding genetic associations with mastitis and the immune system to actually improve disease resistance. This is not necessarily a straight forward question given the plethora of genes, including their additive, dominant, epistatic, and epigenetic interactions. It is sometimes possible to make genetic gains in livestock health to a particular disease by selecting for or against a specific gene. Some examples of this include selection against Mareks Disease of poultry based on MHC haplotypes (64), bovine dermatopholosis using information on BoLA (65), brachyspina in cattle (66) among others (67). It is generally straightforward to make genetic gains for diseases caused by single recessive disorders, whereas information on single genes or clusters of genes may be less informative when trying to enhance resistance to complex traits, such as mastitis resistance, which is caused by a diverse set of pathogens controlled by a large variety of genes and gene interactions (68).

It is also worth noting that the immune system, which is the body’s main host defense system, is regulated by thousands of genes (69). This points to the critical importance and complex nature of disease resistance as an overall fitness trait (70, 71). In fact, recent information from a human systems biology data base on immunity known as the immunogenetic-related information source – IRIS provides evidence for 1,535 immune response genes as of April 2013^1^. This list of genes was curated by IRIS with the following strict definition of a bona fide immune response gene, “a complete gene that produces a functional transcript and demonstrates at least one of the following defense characteristics: (i) known or putative function in innate or adaptive immunity, (ii) participates in the development or maturation of immune system components, (iii) induced by immunomodulators, (iv) encodes a protein expressed primarily in immune tissues, (v) participates in an immune pathway that results in the expression of defense molecules, (vi) produces a protein that interacts directly with pathogens or their products”^2^. When a broader definition of immune response genes are given that seeks to retrieve all genes that have some immune system or related functions, such as that provided by the Immunology Database and Analysis Portal (ImmPort), the list of genes is in the range of 6000^3^. Although these databases are based on human genes the newest version of the innate immunity database, InnateDB, does incorporate a list of bovine genes, including pathway and molecular interactions^4^. As pointed out by Karin Breuer and colleagues, as the experimental data from cattle research validates genetic interactions and immunological pathways this will allow for a deepened understanding of important bovine diseases, such as mastitis and tuberculosis (69). At the moment, these immunological databases rely largely on orthological-based approach to predict pathways. As of September 2012, the InnateDB contained more than 70,000 bovine interactions based on orthology and pathway analysis could assign to more than 7000 bovine genes (69). However, since the bovine immune system does contain some unique genetic features, such as a novel bovine type 1 interferon family known as IFNX, it will not always suffice to rely on orthogues from other species. Nonetheless, it is interesting to speculate about similar genetic pathways. For example, work in human beings has shown that following exposure to bacterial endotoxin a set of 3,714 unique genes were differentially expressed. These changes in genes of interest were confirmed in follow-up microarray experiments (72). Similar transcriptional changes might be predicted in cattle exposed to endotoxin from *E. coli* following intramammary exposure (73), as the complex plethora of genes involved in response to mastitis, such as that caused by *E. coli* is well known (74–76). The goal of this type of systems biology research is to provide a portrait of the entire “interactome between the innate and adaptive immune system, as well as its interconnection with other body systems in the hopes to enhance disease prevention and treatment strategies.

## Genetic Selection for Disease Resistance

Current genetic selection approaches to improve mastitis resistance include both direct and indirect methods. With the exception of Nordic countries that have been selecting for disease resistance for over 35 years (77), most countries breed for mastitis resistance indirectly through SCC (78). More recently, France (79) and Canada (80) have launched routine national genetic and genomic evaluations for clinical mastitis. Problems associated with breeding directly for mastitis resistance include low heritability, the need for accurate health recording, and perhaps most importantly, the potential to skew the immune system causing individuals to be susceptible to other harmful pathogens. This skewing is thought to occur since antibody and cell-mediated immune responses are independent or slightly negatively correlated traits (81–84). This means that improvement for one of these traits does not translate into improvement of the other adaptive immune response trait. This concept will be discussed in more detail.

The heritability of mastitis resistance is low, with estimates ranging from about 0.02–0.10 (85, 86). SCS is genetically correlated (0.7) with mastitis and has a higher heritability of about 0.17, which is why it is used as an alternative trait to breed for resistance to mastitis (87–89). Divergent selection experiments based on SCS in sheep and cattle have been performed with the goal of creating lines of animals with an ability to resist intramammary infection (90, 91). Although these studies have shown a decrease in mastitis in the low SCS line, caution must be used in this approach to improve udder health. SCS tends to monitor subclinical cases (92) and although decreasing bulk tank counts has been associated with a decline in subclinical mastitis; clinical mastitis continues to be a problem (93). Further, since the cells that constitute the SCS are cells of the immune system, too low a SCS has been associated with an increased risk of clinical mastitis (94). In Canada, the approach will be to equally weight clinical mastitis and SCS in the LPI starting in August 2014. Other immune response traits known to associate with resistance to various diseases, including mastitis, may be added subsequently, although sires with improved immune responses are already available through the Canadian breeding company, the Semex Alliance since December 2012 (32).

In order to select directly for mastitis resistance, accurate disease records are essential. Many countries record disease on a voluntary basis, as is the current situation in the United States (86, 95) and Canada (85, 96). The use of voluntary producer records has brought into question the reliability of the estimates for disease resistance. By applying minimum lactation incidence rates to producer-recorded data to include only herds with regular recording, it has been found that although the heritability of disease resistance tends to be low (0.01–0.20) significant genetic variation exists to select for disease resistance (85, 95–97). Some research has demonstrated the use of genomics to improve the reliability of genetic estimates for disease resistance traits (86).

Selection against clinical mastitis has the potential to leave cattle susceptible to infection with other mastitis pathogens, since bacteria require unique immune responses for host protection (2), and mastitis pathogens have been demonstrated to change over time and geographically (7). Further, mastitis-causing pathogens tend to be extracellular in nature, requiring robust antibody responses (98). Since antibody- and cell-mediated immune responses tend to be negatively genetically correlated (83, 84) selection for mastitis resistance may potentially leave individuals with diminished capability to respond to intracellular pathogens generally controlled by the cell-mediated immune response. Cell-mediated responses have been demonstrated to be critical in controlling *Mycobacterium avium* spp *tuberculosis*, the causative pathogen associated with Johne’s disease in cattle (99). Maintaining balanced immune responsiveness is an essential consideration in any breeding program to improve animal health. The other contributing factor is that different BoLA alleles have been shown to associate with antibody versus cell-mediated immune responses, as well as mastitis resistance (41). However, these are not the same alleles that associate with resistance to other viral or parasitic pathogens (100, 101). Therefore, caution must be exercised when selecting for resistance to one specific disease, particularly when it can be caused by multiple pathogens, as is the case with mastitis. Nonetheless, mastitis is such a costly disease that it is likely to be included in selection indices in conjunction with other health traits, such as SCS, until alternative approaches based on optimizing host defense mechanisms are more widely available. For example, in Canada an index for mastitis resistance was developed that includes both clinical mastitis and SCS traits and will be added to the Lifetime Profitability Index (LPI) in August 2014 (102, 103).

**Figure 1:**
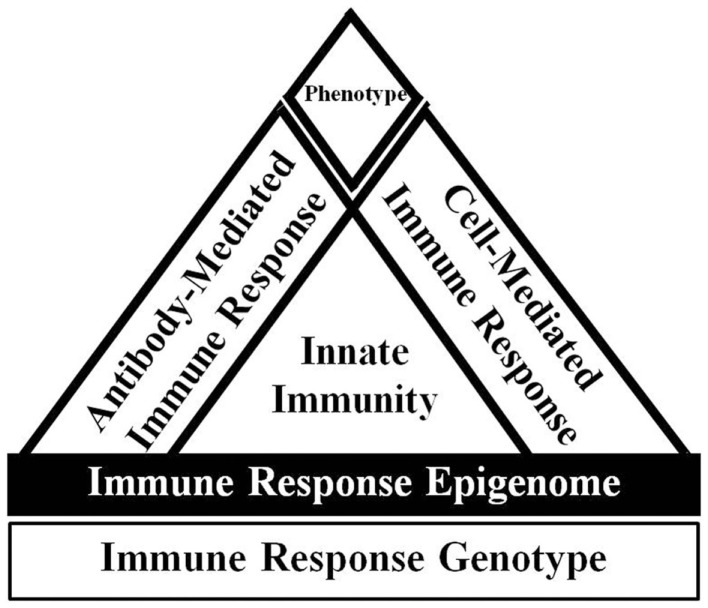
**Overview of immune response adapted from Ref. (105)**. The host immune response phenotype is ultimately determined by the interaction of the immune response genotype with the environment. The expression of the immune response genotype is also regulated by epigenetic effects. The innate immune response is relatively fast acting and non-specific, but is critical to signal appropriate adaptive cell-mediated and antibody-mediated immune responses. Dairy cattle with enhanced and balanced cell and antibody-mediated immune responses are known as high immune responders.

A combination of approaches is likely necessary to decrease mastitis occurrence, such as breeding for broad-based disease resistance based on immune response traits. Breeding for enhanced immune responsiveness is a solution to provide cows with an overall superior ability to respond to a variety of pathogen types requiring unique responses to provide broad-based disease resistance. Individuals with greater and optimally balanced antibody and cell-mediated immune responses breeding values are referred to as high immune responders (HIR) (Figure 1) and the method for identifying such individuals is referred to as the HIR technology (32, 104).

The HIR technology has been used to identify the ability of cows, calves, and bulls to mount antibody and cell-mediated adaptive immune responses (106, 107). These adaptive immune response traits are heritable, on average 0.25–0.35 (83, 84), considerably higher than estimates for specific clinical or subclinical disease resistance (Table 1). The heritability of immune response is similar to what has been found for milk production traits, indicating it would be possible to make significant genetic gain depending on how heavily health is weighted within the selection index. Cows with superior adaptive immune responses have been demonstrated to have substantially lower occurrence of diseases, including mastitis, metritis, displaced abomasums, retained fetal membranes (108) and are less likely to be seropositive for *Mycobacterium avium* spp *paratuberculosis* (109). It would, therefore, be feasible and desirable to breed dairy cows for enhanced immune responses to decrease the occurrence of diseases like mastitis (100, 110). Previously, this approach was shown to improve disease resistance of pigs (105). It should also be noted that producing robust adaptive immune responses requires appropriate priming via particular innate host defense pathways, such as TLR signaling (37). Priming the immune system with LPS in the udder has been shown to reduce bacterial load in experimentally induced mastitis via the TLR signaling (111, 112).

**Table 1:** **Heritability estimates of immune response, mastitis resistance, and milk production and in Holstein dairy cattle**.

Trait	Heritability	Reference
Antibody-mediated immune response	0.16–0.42	Heriazon et al. (84), Thompson-Crispi et al. (83)
Cell-mediated immune response	0.19–0.43	Heriazon et al. (84), Thompson-Crispi et al. (83)
Generalized immunity	0.21	Abdel-Azim et al. (113)
Mastitis	0.02–0.10	Bloemhof et al. (87), Koeck et al. (85, 114), Parker Gaddis et al. (86), Pritchard et al. (115)
Somatic cell score	0.11–0.17	Bloemhof et al. (87), Jamrozik and Schaeffer (88), Koeck et al. (85, 114), Pritchard et al. (115)
Milk yield (305 days)	0.14–0.30	McCarthy and Veerkamp (116), Pritchard et al. (115)

High immune responding cows have also been found to have an increased response to commercial *E. coli J5* mastitis vaccination (117), as well as improved colostrum quality as measured by specific antibody (117), total immunoglobulin, lactoferrin, and β-lactoglobulin (118). Differences in leukocyte populations between high and low immune responders have also been described, such that cows with superior antibody responses have a higher proportion of B cells in peripheral blood in response to immunization, whereas cows with high cell-mediated responses have a higher baseline proportion of gamma delta (γδ) T cells (119). These differences in the diverse phenotypes identified using the HIR technology suggest potential mechanisms that contribute to decreased disease occurrence among high immune responding individuals.

Multiple studies over many years have found beneficial associations between antibody responses and a lower occurrence of mastitis. A study that evaluated antibody-mediated immune responses to a specified test antigen found cows with superior antibody responses had lower occurrence of mastitis in two out of three herds tested (117). Subsequently, cows with greater antibody responses in a commercial herd in Florida were found to be 1.6–2.5 times less likely to get clinical mastitis compared to other cows in the herd (108, 120). Most recently, a nation-wide study in Canada evaluating the incidence rate of clinical mastitis over a 2-year study period found cows with superior antibody responses to have an incidence rate of 17.1 cases of clinical mastitis/100 cow years compared to average and low responding cows with 27.9 and 30.7 cases, respectively. The low responding cows were also found to have more severe mastitis compared to cows with better immune responses (98). Antibody-mediated immune responses have also been beneficially genetically correlated with some reproductive traits as well as longevity, suggesting that cows with better immune responsiveness and therefore, less disease remain in the herd longer (83).

Conversely, cows with greater cell-mediated immune responses have been found to be less likely to be seropositive for *Mycobacteria avium paratuberculosis* (109). Cell-mediated immune responses are also critical to provide protection against *S. aureus* small colony variants that can cause mastitis and have the ability to survive within host cells (6, 21). Antibody and cell-mediated immune responses have been found to be negatively genetically correlated (83, 84). Consequently, in order to ensure protection to a broad range of pathogens it is essential to identify and select individuals with the capacity to generate both effective antibody and cell-mediated immune responses (32).

The Semex Alliance utilizes the HIR Technology to identify dairy sires with superior immune responsiveness, termed *Immunity*^+^™. Daughters of *Immunity*^+^™ sires have been found to have lower disease occurrence and higher profitability compared to daughters of sires with either an unknown or an average or low immune response type. Specifically, daughters of *Immunity*^+^™ sires in a large herd in the US had a 44% reduction in mastitis, 25% less calf pneumonia, and an 8.5% reduction in all diseases in first lactation heifers (32). These results highlight the benefit and potential to improve disease resistance, in particular mastitis resistance, by improving overall immune responsiveness.

Genomic selection has allowed for the opportunity to include new phenotypes in breeding objectives, particularly those that may be relatively expensive to measure (121). Genomic selection refers to breeding decisions based on genomic estimated breeding values (GEBV), which are calculated using the joint effects of SNP markers across the entire genome (122–124). Using a large reference population with accurate phenotype information, the SNP or haplotype effects for a given trait are estimated. In subsequent generations, only information on the SNP or haplotypes are required to calculate the GEBV (123). Genomic selection has provided many substantial benefits to the dairy industry. Perhaps the most highlighted benefit is in the significant increase in the rate of genetic gain by decreasing generation interval, increasing, and selection intensity the accuracy of estimates (122).

The sequencing of the bovine genome and release of SNP arrays used for genomic selection has led to increases in the genome-wide association studies (GWAS). Many GWAS have been performed, which has lead to the identification of quantitative trait loci or SNP profiles associated with resistance or susceptibility to mastitis (125), or SCC as an indicator of mastitis (126–128). Using the approach, many genes involved in immune response have been found, including cytokines IL-4 and IL-13 as well as IL-17 (129). Recently, a series of GWAS have been performed for general immune responsiveness in dairy cattle and results have been validated in dairy sires (46). Results of this work have identified many genes associated with immune responses including the bovine MHC, the complement systems as well as cytokines including IL-17 and TNF in the genetic regulation of bovine immune system. Results of these GWAS on mastitis resistance and immune response suggest that it is possible to calculate GEBV for mastitis or immune response traits increasing the accuracy of estimates for genetic selection. The next critical steps are to create large reference populations with genotypes and accurate phenotypes for disease and immune response traits in order to improve dairy cattle health.

## Conclusion

The ideal solutions to improve resistance to mastitis are likely to be those that focus on a large number of genes, by using information from GWAS, or selection based on breeding values of immune responses, which take into account complex genetic interactions between the innate and adaptive host defense mechanisms without the necessity of knowing all about each individual gene. Using selection indices also offers the advantage of being able to easily adjust the weights given to the various traits within the index as the selection proceeds. These two approaches may be best suited to help alleviate mastitis, at least until we gain more knowledge about genetic and epigenetic regulation of host defense mechanisms.

## Conflict of Interest Statement

The authors declare that the research was conducted in the absence of any commercial or financial relationships that could be construed as a potential conflict of interest.

## Footnotes

^1^http://www.innatedb.com/curatedGenes

^2^http://www.innatedb.com/redirect.do?go=resourcesGeneLists

^3^http://www.immport.org

^4^http://www.innatedb.com

## References

[1] De Vliegher S Fox LK Piepers S McDougall S Barkema HW Invited review: mastitis in dairy heifers: nature of the disease, potential impact, prevention, and control. J Dairy Sci (2012) 95:1025–40 10.3168/jds.2010-4074 22365187

[2] Schukken YH Gunther J Fitzpatrick J Fontaine MC Goetze L Holst O Host-response patterns of intramammary infections in dairy cows. Vet Immunol Immunopathol (2011) 144:270–89 10.1016/j.vetimm.2011.08.022 21955443

[3] Aitken SL Corl CM Sordillo LM Immunopathology of mastitis: insights into disease recognition and resolution. J Mammary Gland Biol Neoplasia (2011) 16:291–304 10.1007/s10911-011-9230-4 21938490

[4] Sordillo LM Shafer-Weaver K DeRosa D Immunobiology of the mammary gland. J Dairy Sci (1997) 80:1851–65 10.3168/jds.S0022-0302(97)76121-6 9276826

[5] Zadoks R Understanding the sources, transmission routes and prognoses for mastitis pathogens. WCDS Adv Dairy Technol (2014) 26:91–100

[6] Atalla H Wilkie B Gyles C Leslie K Mutharia L Mallard B Antibody and cell-mediated immune responses to *Staphylococcus aureus* small colony variants and their parental strains associated with bovine mastitis. Dev Comp Immunol (2010) 34:1283–90 10.1016/j.dci.2010.07.005 20670645

[7] Bradley A Bovine mastitis: an evolving disease. Vet J (2002) 164:116–28 10.1053/tvjl.2002.0724 12359466

[8] Contreras GA Rodriguez JM Mastitis: comparative etiology and epidemiology. J Mammary Gland Biol Neoplasia (2011) 16:339–56 10.1007/s10911-011-9234-0 21947764

[9] Kehrli ME Jr Shuster DE Factors affecting milk somatic cells and their role in health of the bovine mammary gland. J Dairy Sci (1994) 77:619–27 10.3168/jds.S0022-0302(94)76992-7 8182187

[10] Steeneveld W Hogeveen H Barkema HW van den BJ Huirne RB The influence of cow factors on the incidence of clinical mastitis in dairy cows. J Dairy Sci (2008) 91:1391–402 10.3168/jds.2007-0705 18349231

[11] Bannerman DD Pathogen-dependent induction of cytokines and other soluble inflammatory mediators during intramammary infection of dairy cows. J Anim Sci (2009) 87:10–25 10.2527/jas.2008-1187 18708595

[12] Wellnitz O Arnold ET Bruckmaier RM Lipopolysaccharide and lipoteichoic acid induce different immune responses in the bovine mammary gland. J Dairy Sci (2011) 94:5405–12 10.3168/jds.2010-3931 22032363

[13] Ballou MA Inflammation: role in the etiology and pathophysiology of clinical mastitis in dairy cows. J Anim Sci (2011) 90:1466–78 10.2527/jas.2011-4663 22079996

[14] Zhao X Lacasse P Mammary tissue damage during bovine mastitis: causes and control. J Anim Sci (2008) 86:57–65 10.2527/jas.2007-0302 17785603

[15] Borghesi L Milcarek C Innate versus adaptive immunity: a paradigm past its prime? Cancer Res (2007) 67:3989–93 10.1158/0008-5472.CAN-07-0182 17483307

[16] Sordillo LM Streicher KL Mammary gland immunity and mastitis susceptibility. J Mammary Gland Biol Neoplasia (2002) 7:135–46 10.1023/A:1020347818725 12463736

[17] Rainard P Riollet C Innate immunity of the bovine mammary gland. Vet Res (2006) 37:369–400 10.1051/vetres:2006007 16611554

[18] Wellnitz O Bruckmaier RM The innate immune response of the bovine mammary gland to bacterial infection. Vet J (2012) 192:148–52 10.1016/j.tvjl.2011.09.013 22498784

[19] Iwasaki A Medzhitov R Regulation of adaptive immunity by the innate immune system. Science (2010) 327:291–5 10.1126/science.1183021 20075244 PMC3645875

[20] Hermann C Review: variability of host–pathogen interaction. J Endotoxin Res (2007) 13:199–217 10.1177/0968051907082605 17956939

[21] Atalla H Gyles C Mallard B Persistence of a *Staphylococcus aureus* small colony variants (*S. aureus* SCV) within bovine mammary epithelial cells. Vet Microbiol (2010) 143:319–28 10.1016/j.vetmic.2009.11.030 20022186

[22] Bishop S Woolliams JA Genomics and disease resistance studies in livestock. Livestock Sci (2014) 166:190–8 10.1016/j.livsci.2014.04.034 PMC4547482 26339300

[23] Walsh C Fanning S Antimicrobial resistance in foodborne pathogens – a cause for concern? Curr Drug Targets (2008) 9:808–15 10.2174/138945008785747761 18781926

[24] Oliver SP Murinda SE Jayarao BM Impact of antibiotic use in adult dairy cows on antimicrobial resistance of veterinary and human pathogens: a comprehensive review. Foodborne Pathog Dis (2011) 8:337–55 10.1089/fpd.2010.0730 21133795

[25] Fitzpatrick CE Chapinal N Petersson-Wolfe CS Devries TJ Kelton DF Duffield TF The effect of meloxicam on pain sensitivity, rumination time, and clinical signs in dairy cows with endotoxin-induced clinical mastitis. J Dairy Sci (2013) 96:2847–56 10.3168/jds.2012-5855 23522672

[26] Bharathan M Mullarky IK Targeting mucosal immunity in the battle to develop a mastitis vaccine. J Mammary Gland Biol Neoplasia (2011) 16:409–19 10.1007/s10911-011-9233-1 21968537

[27] Oviedo-Boyso J Valdez-Alarcon JJ Cajero-Juarez M Ochoa-Zarzosa A Lopez-Meza JE Bravo-Patino A Innate immune response of bovine mammary gland to pathogenic bacteria responsible for mastitis. J Infect (2007) 54:399–409 10.1016/j.jinf.2006.06.010 16882453

[28] Strandberg Y Gray C Vuocolo T Donaldson L Broadway M Tellam R Lipopolysaccharide and lipoteichoic acid induce different innate immune responses in bovine mammary epithelial cells. Cytokine (2005) 31:72–86 10.1016/j.cyto.2005.02.010 15882946

[29] Brenaut P Lefevre L Rau A Laloe D Pisoni G Moroni P Contribution of mammary epithelial cells to the immune response during early stages of a bacterial infection to *Staphylococcus aureus*. Vet Res (2014) 45:16 10.1186/1297-9716-45-16 24521038 PMC3937043

[30] Schwarz D Rivas AL Konig S Diesterbeck US Schlez K Zschock M CD2/CD21 index: a new marker to evaluate udder health in dairy cows. J Dairy Sci (2013) 96:5106–19 10.3168/jds.2013-6804 23769358

[31] Kandasamy S Green BB Benjamin AL Kerr DE Between-cow variation in dermal fibroblast response to lipopolysaccharide reflected in resolution of inflammation during *Escherichia coli* mastitis. J Dairy Sci (2011) 94:5963–75 10.3168/jds.2011-4288 22118085

[32] Mallard BA Cartwright S Emam M Fleming K Gallo N Hodgins DC Genetic selection of cattle for improved immunity and health. WCDS Adv Dairy Technol (2014) 26:247–57

[33] Green BB Kerr DE Epigenetic contribution to individual variation in response to lipopolysaccharide in bovine dermal fibroblasts. Vet Immunol Immunopathol (2014) 157:49–58 10.1016/j.vetimm.2013.10.015 24268632 PMC4228796

[34] Paibomesai M Hussey B Nino-Soto M Mallard BA Effects of parturition and dexamethasone on DNA methylation patterns of IFN-gamma and IL-4 promoters in CD4^+^ T-lymphocytes of Holstein dairy cows. Can J Vet Res (2013) 77:54–62 23814356 PMC3525172

[35] Werling D Piercy J Coffey TJ Expression of TOLL-like receptors (TLR) by bovine antigen-presenting cells-potential role in pathogen discrimination? Vet Immunol Immunopathol (2006) 112:2–11 10.1016/j.vetimm.2006.03.007 16701904

[36] Akira S Mammalian toll-like receptors. Curr Opin Immunol (2003) 15:5–11 10.1016/S0952-7915(03)00005-0 12495726

[37] Reuven EM Fink A Shai Y Regulation of innate immune responses by transmembrane interactions: lessons from the TLR family. Biochim Biophys Acta (2014) 1838:1586–93 10.1016/j.bbamem.2014.01.020 24480409

[38] Petzl W Zerbe H Gunther J Yang W Seyfert HM Nurnberg G *Escherichia coli*, but not *Staphylococcus aureus* triggers an early increased expression of factors contributing to the innate immune defense in the udder of the cow. Vet Res (2008) 39:18 10.1051/vetres:2007057 18258172

[39] Ellis SA Codner G The impact of MHC diversity on cattle T cell responses. Vet Immunol Immunopathol (2012) 148:74–7 10.1016/j.vetimm.2011.03.009 21466899

[40] Mallard BA Leslie KE Dekkers JC Hedge R Bauman M Stear MJ Differences in bovine lymphocyte antigen associations between immune responsiveness and risk of disease following intramammary infection with *Staphylococcus aureus*. J Dairy Sci (1995) 78:1937–44 10.3168/jds.S0022-0302(95)76819-9 8550903

[41] Rupp R Hernandez A Mallard BA Association of bovine leukocyte antigen (BoLA) DRB3.2 with immune response, mastitis, and production and type traits in Canadian Holsteins. J Dairy Sci (2007) 90:1029–38 10.3168/jds.S0022-0302(07)71589-8 17235182

[42] Sharif S Mallard BA Wilkie BN Sargeant JM Scott HM Dekkers JC Associations of the bovine major histocompatibility complex DRB3 (BoLA-DRB3) alleles with occurrence of disease and milk somatic cell score in Canadian dairy cattle. Anim Genet (1998) 29:185–93 10.1111/j.1365-2052.1998.00318.x 9720177

[43] Yoshida T Furuta H Kondo Y Mukoyama H Association of BoLA-DRB3 alleles with mastitis resistance and susceptibility in Japanese Holstein cows. Anim Sci J (2012) 83:359–66 10.1111/j.1740-0929.2011.00972.x 22574787

[44] Chu MX Ye SC Qiao L Wang JX Feng T Huang DW Polymorphism of exon 2 of BoLA-DRB3 gene and its relationship with somatic cell score in Beijing Holstein cows. Mol Biol Rep (2012) 39:2909–14 10.1007/s11033-011-1052-3 21687974

[45] Pashmi M Qanbari S Ghorashi SA Sharifi AR Simianer H Analysis of relationship between bovine lymphocyte antigen DRB3.2 alleles, somatic cell count and milk traits in Iranian Holstein population. J Anim Breed Genet (2009) 126:296–303 10.1111/j.1439-0388.2008.00783.x 19630880

[46] Thompson-Crispi KA Sargolzaei M Ventura R Abo-Ismail M Miglior F Schenkel F A genome-wide association study for immune response traits in Canadian Holstein cattle. BMC Genomics (2014) 15:559 10.1186/1471-2164-15-559 24996426 PMC4099479

[47] de Mesquita AQ CS ER de Mesquita AJ Jardim EA Kipnis AP Association of TLR4 polymorphisms with subclinical mastitis in Brazilian holsteins. Braz J Microbiol (2012) 43:692–7 10.1590/S1517-83822012000200034 24031881 PMC3768839

[48] Sharma BS Mount J Karrow NA Functional characterization of a single nucleotide polymorphism in the 5’ UTR region of the bovine toll-like receptor 4 gene. Dev Biol (Basel) (2008) 132:331–6 10.1159/000317179 18817322

[49] Pant SD Schenkel FS Leyva-Baca I Sharma BS Karrow NA Identification of polymorphisms in bovine TLR2 and CARD15, associations between CARD15 polymorphisms and milk somatic cell score in Canadian Holsteins, and functional relevance of SNP c.3020A >T. Dev Biol (Basel) (2008) 132:247–53 10.1159/000317167 18817309

[50] Verschoor CP Pant SD Schenkel FS Sharma BS Karrow NA SNPs in the bovine IL-10 receptor are associated with somatic cell score in Canadian dairy bulls. Mamm Genome (2009) 20:447–54 10.1007/s00335-009-9198-1 19641966

[51] Alain K Karrow NA Thibault C St-Pierre J Lessard M Bissonnette N Osteopontin: an early innate immune marker of *Escherichia coli* mastitis harbors genetic polymorphisms with possible links with resistance to mastitis. BMC Genomics (2009) 10:444 10.1186/1471-2164-10-444 19765294 PMC2761946

[52] Galvao KN Pighetti GM Cheong SH Nydam DV Gilbert RO Association between interleukin-8 receptor-alpha (CXCR1) polymorphism and disease incidence, production, reproduction, and survival in Holstein cows. J Dairy Sci (2011) 94:2083–91 10.3168/jds.2010-3636 21426999

[53] Leyva-Baca I Schenkel F Martin J Karrow NA Polymorphisms in the 5′ upstream region of the CXCR1 chemokine receptor gene, and their association with somatic cell score in Holstein cattle in Canada. J Dairy Sci (2008) 91:407–17 10.3168/jds.2007-0142 18096965

[54] Verbeke J Piepers S Peelman L Van PM De VS Pathogen-group specific association between CXCR1 polymorphisms and subclinical mastitis in dairy heifers. J Dairy Res (2012) 79:341–51 10.1017/S0022029912000349 22850581

[55] Leyva-Baca I Schenkel F Sharma BS Jansen GB Karrow NA Identification of single nucleotide polymorphisms in the bovine CCL2, IL8, CCR2 and IL8RA genes and their association with health and production in Canadian Holsteins. Anim Genet (2007) 38:198–202 10.1111/j.1365-2052.2007.01588.x 17433017

[56] Pighetti GM Elliott AA Gene polymorphisms: the keys for marker assisted selection and unraveling core regulatory pathways for mastitis resistance. J Mammary Gland Biol Neoplasia (2011) 16:421–32 10.1007/s10911-011-9238-9 21997401

[57] Meade KG Cormican P Narciandi F Lloyd A O’Farrelly C Bovine beta-defensin gene family: opportunities to improve animal health? Physiol Genomics (2014) 46:17–28 10.1152/physiolgenomics.00085.2013 24220329

[58] Karrow NA Sharma BS Fisher RE Mallard BA Epigenetics and animal health. Comprehensive Biotechonology. 2nd ed Waltham, MA: Elsevier B.V (2011). p. 381–94 10.1016/B978-0-08-088504-9.00289-0

[59] Wilson CB Rowell E Sekimata M Epigenetic control of T-helper-cell differentiation. Nat Rev Immunol (2009) 9:91–105 10.1038/nri2487 19151746

[60] Zhu J Yamane H Paul WE Differentiation of effector CD4 T cell populations (*). Annu Rev Immunol (2010) 28:445–89 10.1146/annurev-immunol-030409-101212 20192806 PMC3502616

[61] Doherty R O’Farrelly C Meade KG Epigenetic regulation of the innate immune response to LPS in bovine peripheral blood mononuclear cells (PBMC). Vet Immunol Immunopathol (2013) 154:102–10 10.1016/j.vetimm.2013.05.004 23764468

[62] Jin W Ibeagha-Awemu EM Liang G Beaudoin F Zhao X Guan LL Transcriptome microRNA profiling of bovine mammary epithelial cells challenged with *Escherichia coli* or *Staphylococcus aureus* bacteria reveals pathogen directed microRNA expression profiles. BMC Genomics (2014) 15:181 10.1186/1471-2164-15-181 24606609 PMC4029070

[63] Lawless N Foroushani AB McCabe MS O’Farrelly C Lynn DJ Next generation sequencing reveals the expression of a unique miRNA profile in response to a gram-positive bacterial infection. PLoS One (2013) 8:e57543 10.1371/journal.pone.0057543 23472090 PMC3589390

[64] Schat KA Taylor RL Jr Briles WE Resistance to Marek’s disease in chickens with recombinant haplotypes to the major histocompatibility (B) complex. Poult Sci (1994) 73:502–8 10.3382/ps.0730502 8202429

[65] Maillard JC Berthier D Chantal I Thevenon S Sidibe I Stachurski F Selection assisted by a BoLA-DR/DQ haplotype against susceptibility to bovine dermatophilosis. Genet Sel Evol (2003) 35(Suppl 1):S193–200 10.1186/1297-9686-35-S1-S193 12927091 PMC3231761

[66] Charlier C Agerholm JS Coppieters W Karlskov-Mortensen P Li W de JG A deletion in the bovine FANCI gene compromises fertility by causing fetal death and brachyspina. PLoS One (2012) 7:e43085 10.1371/journal.pone.0043085 22952632 PMC3430679

[67] Charlier C Coppieters W Rollin F Desmecht D Agerholm JS Cambisano N Highly effective SNP-based association mapping and management of recessive defects in livestock. Nat Genet (2008) 40:449–54 10.1038/ng.96 18344998

[68] Rupp R Boichard D Genetics of resistance to mastitis in dairy cattle. Vet Res (2003) 34:671–88 10.1051/vetres:2003020 14556700

[69] Breuer K Foroushani AK Laird MR Chen C Sribnaia A Lo R InnateDB: systems biology of innate immunity and beyond. Nucleic Acid Res (2013) 41:D1228–33 10.1093/nar/gks1147 23180781 PMC3531080

[70] Berry DP Bermingham ML Good M More SJ Genetics of animal health and disease in cattle. Ir Vet J (2011) 64:5 10.1186/2046-0481-64-5 21777492 PMC3102331

[71] Gibson JP Bishop SC Use of molecular markers to enhance resistance of livestock to disease: a global approach. Rev Sci Tech (2005) 24:343–53 16110901

[72] Calvano SE Xiao W Richards DR Felciano RM Baker HV Cho RJ A network-based analysis of systemic inflammation in humans. Nature (2005) 437:1032–7 10.1038/nature03985 16136080

[73] Buitenhuis B Røntved CM Edwards SM Ingvartsen KL Sørensen P In depth analysis of genes and pathways of the mammary gland involved in the pathogenesis of bovine *Escherichia coli*-mastitis. BMC Genomics (2011) 28(12):130 10.1186/1471-2164-12-130 21352611 PMC3053262

[74] Gunther J Koczan D Yang W Nurnberg G Repsilber D Schuberth HJ Assessment of the immune capacity of mammary epithelial cells: comparison with mammary tissue after challenge with *Escherichia coli*. Vet Res (2009) 40:31 10.1051/vetres/2009014 19321125 PMC2695127

[75] Mitterhuemer S Petzl W Krebs S Mehne D Klanner A Wolf E *Escherichia coli* infection induces distinct local and systemic transcriptome responses in the mammary gland. BMC Genomics (2010) 11:138 10.1186/1471-2164-11-138 20184744 PMC2846913

[76] Jensen K Gunther J Talbot R Petzl W Zerbe H Schuberth HJ *Escherichia coli*- and *Staphylococcus aureus*-induced mastitis differentially modulate transcriptional responses in neighbouring uninfected bovine mammary gland quarters. BMC Genomics (2013) 14:36 10.1186/1471-2164-14-36 23324411 PMC3598231

[77] Osteras O Solbu H Refsdal AO Roalkvam T Filseth O Minsaas A Results and evaluation of thirty years of health recordings in the Norwegian dairy cattle population. J Dairy Sci (2007) 90:4483–97 10.3168/jds.2007-0030 17699070

[78] Miglior F Muir BL Van Doormaal BJ Selection indices in Holstein cattle of various countries. J Dairy Sci (2005) 88:1255–63 10.3168/jds.S0022-0302(05)72792-2 15738259

[79] Govignon-Gion A Dassonneville R Baloche G Ducrocq V Genetic evaluation of mastitis in dairy cattle in France. Interbull Bull (2012). Available from: https://journal.interbull.org/index.php/ib/article/view/1276/1314, 10.1017/S1751731115002529 26592099

[80] Jamrozik J Koeck A Miglior F Kistemaker G Schenkel F Kelton D Genetic and genomic evaluation of mastitis resistance in Canada. Interbull Bull (2013). Available from: https://journal.interbull.org/index.php/ib/article/view/1291/1365,

[81] Mouton D Bouthillier Y Mevel JC Biozzi G Genetic selection for antibody responsiveness in mice: further evidence for inverse modification of macrophage catabolic activity without alteration of the expression of T-cell-mediated immunity. Ann Immunol (1984) 135D:173–86 6393858 10.1016/s0769-2625(84)81109-5

[82] Mallard BA Wilkie BN Kennedy BW Quinton M Use of estimated breeding values in a selection index to breed Yorkshire pigs for high and low immune and innate resistance factors. Anim Biotechnol (1992) 3:257–80 10.1080/10495399209525776

[83] Thompson-Crispi KA Sewalem A Miglior F Mallard B Genetic parameters of adaptive immune response traits in Canadian Holsteins. J Dairy Sci (2012) 95:401–9 10.3168/jds.2011-4452 22192219

[84] Heriazon A Quinton M Miglior F Leslie KE Sears W Mallard BA Phenotypic and genetic parameters of antibody and delayed-type hypersensitivity responses of lactating Holstein cows. Vet Immunol Immunopathol (2013) 154:83–92 10.1016/j.vetimm.2013.03.014 23747204

[85] Koeck A Miglior F Kelton DF Schenkel FS Health recording in Canadian Holsteins: data and genetic parameters. J Dairy Sci (2012) 95:4099–108 10.3168/jds.2011-5127 22720966

[86] Parker Gaddis KL Cole JB Clay JS Maltecca C Genomic selection for producer-recorded health event data in US dairy cattle. J Dairy Sci (2014) 97:3190–9 10.3168/jds.2013-7543 24612803

[87] Bloemhof S de Jong G de Haas Y Genetic parameters for clinical mastitis in the first three lactations of Dutch Holstein cattle. Vet Microbiol (2008) 134:165–71 10.1016/j.vetmic.2008.09.024 18945557

[88] Jamrozik J Schaeffer LR Test-day somatic cell score, fat-to-protein ratio and milk yield as indicator traits for sub-clinical mastitis in dairy cattle. J Anim Breed Genet (2012) 129:11–9 10.1111/j.1439-0388.2011.00929.x 22225580

[89] Koeck A Miglior F Kelton DF Schenkel FS Alternative somatic cell count traits to improve mastitis resistance in Canadian Holsteins. J Dairy Sci (2012) 95:432–9 10.3168/jds.2011-4731 22192222

[90] Brand B Hartmann A Repsilber D Griesbeck-Zilch B Wellnitz O Kuhn C Comparative expression profiling of *E. coli* and *S. aureus* inoculated primary mammary gland cells sampled from cows with different genetic predispositions for somatic cell score. Genet Sel Evol (2011) 43:24 10.1186/1297-9686-43-24 21702919 PMC3143085

[91] Rupp R Bergonier D Dion S Hygonenq MC Aurel MR Robert-Granie C Response to somatic cell count-based selection for mastitis resistance in a divergent selection experiment in sheep. J Dairy Sci (2009) 92:1203–19 10.3168/jds.2008-1435 19233814

[92] Barkema HW Schukken YH Lam TJ Beiboer ML Wilmink H Benedictus G Incidence of clinical mastitis in dairy herds grouped in three categories by bulk milk somatic cell counts. J Dairy Sci (1998) 81:411–9 10.3168/jds.S0022-0302(98)75591-2 9532494

[93] Olde Riekerink RG Barkema HW Kelton DF Scholl DT Incidence rate of clinical mastitis on Canadian dairy farms. J Dairy Sci (2008) 91:1366–77 10.3168/jds.2007-0757 18349229

[94] Suriyasathaporn W Schukken YH Nielen M Brand A Low somatic cell count: a risk factor for subsequent clinical mastitis in a dairy herd. J Dairy Sci (2000) 83:1248–55 10.3168/jds.S0022-0302(00)74991-5 10877390

[95] Zwald NR Weigel KA Chang YM Welper RD Clay JS Genetic selection for health traits using producer-recorded data. I. Incidence rates, heritability estimates, and sire breeding values. J Dairy Sci (2004) 87:4287–94 10.3168/jds.S0022-0302(04)73573-0 15545392

[96] Neuenschwander TF Miglior F Jamrozik J Berke O Kelton DF Schaeffer LR Genetic parameters for producer-recorded health data in Canadian Holstein cattle. Animal (2012) 6:571–8 10.1017/S1751731111002059 22436272

[97] Heringstad B Chang YM Gianola D Klemetsdal G Genetic analysis of clinical mastitis, milk fever, ketosis, and retained placenta in three lactations of Norwegian red cows. J Dairy Sci (2005) 88:3273–81 10.3168/jds.S0022-0302(05)73010-1 16107417

[98] Thompson-Crispi KA Miglior F Mallard BA Incidence rates of clinical mastitis among Canadian Holsteins classified as high, average and low immune responders. Clin Vaccine Immunol (2013) 20:106–12 10.1128/CVI.00494-12 23175290 PMC3535773

[99] Begg DJ de SK Carter N Plain KM Purdie A Whittington RJ Does a Th1 over Th2 dominancy really exist in the early stages of *Mycobacterium avium* subspecies *paratuberculosis* infections? Immunobiology (2011) 216:840–6 10.1016/j.imbio.2010.12.004 21281979

[100] Stear MJ Bishop SC Mallard BA Raadsma H The sustainability, feasibility and desirability of breeding livestock for disease resistance. Res Vet Sci (2001) 71:1–7 10.1053/rvsc.2001.0496 11666141

[101] Zanotti M Poli G Ponti W Polli M Rocchi M Bolzani E Association of BoLA class II haplotypes with subclinical progression of bovine leukaemia virus infection in Holstein-Friesian cattle. Anim Genet (1996) 27:337–41 8930075

[102] Miglior F Koeck A Jamrozik J Schenkel FS Kelton DF Kistemaker GJ Index for mastitis resistance and use of BHBA for evaluation of health traits in Canadian Holsteins. Interbull Bull (2014). Available online https://journal.interbull.org/index.php/ib/article/view/1349/1420,

[103] Van Doormaal B Beavers L Mastitis Resistance Selection: Now a Reality!. (2014). Available from: http://www.cdn.ca/articles.php

[104] Wagter-Lesperance L Cartwright S Funk T Kelton D Miglior F Mallard B Feasibility of high immune response (HIR) technology as a health management tool to characterize immune response profiles of dairy cattle. In: Hogeveen H Lam TJ, editors. Udder Health and Communication. Wageningen: Wageningen Academic Publishers (2012). p. 359–66

[105] Wilkie B Mallard B Selection for high immune response: an alternative approach to animal health maintenance? Vet Immunol Immunopathol (1999) 72:231–5 10.1016/S0165-2427(99)00136-1 10614513

[106] Cartwright SL Begley N Schaeffer LR Burnside EB Mallard BA Antibody and cell-mediated immune responses and survival between Holstein and Norwegian Red x Holstein Canadian calves. J Dairy Sci (2011) 94:1576–85 10.3168/jds.2010-3502 21338823

[107] Thompson-Crispi KA Mallard BA Type 1 and Type 2 immune response profiles of commercial dairy cows in four regions of Canada. Can J Vet Res (2012) 76:120–8 23024454 PMC3314434

[108] Thompson-Crispi KA Hine B Quinton M Miglior F Mallard BA Short communication: association of disease incidence and adaptive immune response in Holstein dairy cows. J Dairy Sci (2012) 95:3888–93 10.3168/jds.2011-5201 22720943

[109] Pinedo PJ Donovan A Rae O DeLapaz J Association between paratuberculosis infection and general immune status in dairy cattle. Proceedings of the 10th International Colloquium on Paratuberculosis, Vol. 1. Minneapolis (2009). p. 127

[110] Mallard BA Atalla H Cartwright S Hine BC Hussey B Paibomesai M Genetic and epigenetic regulation of the bovine immune system: practical implications of the high immune response technology. Proceedings of National Mastitis Council 50th Annual Meeting Verona, WI (2011). 53–63

[111] Gunther J Petzl W Zerbe H Schuberth HJ Koczan D Goetze L Lipopolysaccharide priming enhances expression of effectors of immune defence while decreasing expression of pro-inflammatory cytokines in mammary epithelia cells from cows. BMC Genomics (2012) 13:17 10.1186/1471-2164-13-17 22235868 PMC3315725

[112] Petzl W Gunther J Pfister T Sauter-Louis C Goetze L von AS Lipopolysaccharide pretreatment of the udder protects against experimental *Escherichia coli* mastitis. Innate Immun (2012) 18:467–77 10.1177/1753425911422407 21990573

[113] Abdel-Azim GA Freeman AE Kehrli ME Jr Kelm SC Burton JL Kuck AL Genetic basis and risk factors for infectious and noninfectious diseases in US Holsteins. I. Estimation of genetic parameters for single diseases and general health. J Dairy Sci (2005) 88:1199–207 10.3168/jds.S0022-0302(05)72786-7 15738253

[114] Koeck A Miglior F Kelton DF Schenkel FS Short communication: genetic parameters for mastitis and its predictors in Canadian Holsteins. J Dairy Sci (2012) 95:7363–6 10.3168/jds.2012-5648 23021760

[115] Pritchard T Coffey M Mrode R Wall E Genetic parameters for production, health, fertility and longevity traits in dairy cows. Animal (2013) 7:34–46 10.1017/S1751731112001401 23031504

[116] McCarthy J Veerkamp RF Estimation of genetic parameters for test-day records of dairy traits in a seasonal calving system. J Dairy Sci (2012) 95:5365–77 10.3168/jds.2011-4706 22916943

[117] Wagter LC Mallard BA Wilkie BN Leslie KE Boettcher PJ Dekkers JC A quantitative approach to classifying Holstein cows based on antibody responsiveness and its relationship to peripartum mastitis occurrence. J Dairy Sci (2000) 83:488–98 10.3168/jds.S0022-0302(00)74908-3 10750107

[118] Fleming K Variation of Bioactive Components in Colostrum and Milk from Canadian Holstein Dairy Cattle Classified as High, Average or Low Immune Responders [MSc Thesis]. University of Guelph (2014). Available from: https://atrium.lib.uoguelph.ca/xmlui/bitstream/handle/10214/8185/Fleming_Kelly_201406_MSc.pdf?sequence=3

[119] Hine BC Cartwright SL Mallard BA Analysis of leukocyte populations in Canadian Holsteins classified as high or low immune responders for antibody- or cell-mediated immune response. Can J Vet Res (2012) 76:149–56 23024458 PMC3314438

[120] DeLapaz J Using Humoral and Cellular Response to Novel Antigens in Periparturent Dairy Cows as a Measure of Genetic Disease Resistance in Dairy Cows [MSc Thesis]. University of Florida (2008). Available from: http://etd.fcla.edu/UF/UFE0022360/delapaz_j.pdf

[121] Boichard D Brochard M New phenotypes for new breeding goals in dairy cattle. Animal (2012) 6:544–50 10.1017/S1751731112000018 22436268

[122] Schaeffer LR Strategy for applying genome-wide selection in dairy cattle. J Anim Breed Genet (2006) 123:218–23 10.1111/j.1439-0388.2006.00595.x 16882088

[123] Goddard ME Hayes BJ Mapping genes for complex traits in domestic animals and their use in breeding programmes. Nat Rev Genet (2009) 10:381–91 10.1038/nrg2575 19448663

[124] Schefers JM Weigel KA Genomic selection in dairy cattle: integration of DNA testing into breeding programs. Anim Front (2012) 2:4–9 10.2527/af.2011-0032

[125] Sodeland M Kent MP Olsen HG Opsal MA Svendsen M Sehested E Quantitative trait loci for clinical mastitis on chromosomes 2, 6, 14 and 20 in Norwegian Red cattle. Anim Genet (2011) 42:457–65 10.1111/j.1365-2052.2010.02165.x 21906097

[126] Meredith BK Berry DP Kearney F Finlay EK Fahey AG Bradley DG A genome-wide association study for somatic cell score using the Illumina high-density bovine beadchip identifies several novel QTL potentially related to mastitis susceptibility. Front Genet (2013) 4:229 10.3389/fgene.2013.00229 24223582 PMC3818585

[127] Minozzi G Nicolazzi EL Strozzi F Stella A Negrini R Jmone-Marsan P Genome wide scan for somatic cell counts in holstein bulls. BMC Proc (2011) 4:S17 10.1186/1753-6561-5-S4-S17 21645296 PMC3108211

[128] Wijga S Bastiaansen JW Wall E Strandberg E de HY Giblin L Genomic associations with somatic cell score in first-lactation Holstein cows. J Dairy Sci (2012) 95:899–908 10.3168/jds.2011-4717 22281354

[129] Lewandowska-Sabat AM Gunther J Seyfert HM Olsaker I Combining quantitative trait loci and heterogeneous microarray data analyses reveals putative candidate pathways affecting mastitis in cattle. Anim Genet (2012) 43:793–9 10.1111/j.1365-2052.2012.02342.x 22497313

